# pH-Dependent Compaction of the Intrinsically Disordered Poly-E Motif in Titin

**DOI:** 10.3390/biology11091302

**Published:** 2022-09-01

**Authors:** Sophia Manukian, Gerrick E. Lindberg, Emily Punch, Sudarshi Premawardhana Dassanayake Mudiyanselage, Matthew J. Gage

**Affiliations:** 1 Department of Chemistry, University of Massachusetts Lowell, Lowell, MA 01854, USA; 2 UMass Movement Center (UMOVE), University of Massachusetts Lowell, Lowell, MA 01854, USA; 3 Department of Applied Physics and Materials Science and Department of Chemistry and Biochemistry, Northern Arizona University, Flagstaff, AZ 86011, USA

**Keywords:** intrinsically disordered proteins, titin, PEVK, circular dichroism, conformational change

## Abstract

**Simple Summary:**

The importance of pH shifts in biological systems is often underappreciated, but they can be a common form of regulation by both activating and inactivating important proteins. Proteins without any regular structure, commonly referred to as intrinsically disordered proteins, are very sensitive to pH changes due to their high number of ionizable amino acids. This study focuses on the role of non-ionizable residues in the negatively charged poly-E motif from the PEVK region of the muscle protein titin. Our results demonstrate that aromatic amino acids and the number of proline residues in the linker regions between charged clusters have the greatest impact on the pH-dependent conformational flexibility of this region. These results provide useful new insights into how the sequence between clusters of charges in proteins can impact the sensitivity of a protein to pH shifts.

**Abstract:**

The conformational sensitivity of intrinsically disordered proteins to shifts in pH due to their high degree of charged residues has been recognized for well over a decade. However, the role of the non-ionizable residues in this pH sensitivity remains poorly understood. Our lab has been investigating the pH sensitivity of the poly-E motifs of the PEVK region of the muscle protein titin, which provides an ideal model system to explore this question. Using a series of 15-amino acid peptides derived from one of the poly-E motif sequences, we have investigated the role of side-chain chemistry in the conformational flexibility of this region. Our results demonstrate that aromatic side chains and proline content are the two variables that most influence pH sensitivity. The introduction of aromatic side chains resulted in a more collapsed structure, even at pH 7, while the removal of prolines resulted in a higher degree of pH sensitivity. These results highlight the importance of considering the impact of non-ionizable residues on IDP function, especially when considering the impact of pH on conformational flexibility.

## 1. Introduction

Intrinsically disordered proteins (IDPs) and intrinsically disordered regions (IDRs) lack a specific, defined 3-dimensional native conformation and, instead, exist as an ensemble of alternate conformations. IDPs and IDRs are a growing class of proteins that are especially prevalent in complex eukaryotic systems [1], playing a variety of roles in biological processes, such as cell signaling and regulation [2]. Due to their abundance, distinct functional properties, and importance to biological systems, IDPs remain an interesting area for research.

Among the unique properties of IDPs is their “turned out” response, where they are likely to become more structured under conditions that would denature most globular proteins [3]. An example of one of these conditions is pH, as many IDPs and IDRs gain structure under acidic or basic conditions. This has been experimentally shown for several proteins, including the histidine-rich protein II [4], pig calpastatin domain I [5], and γ-tubulin (C-terminus) [6]. In addition, comprehensive studies have been completed on both α-synuclein [7,8] and prothymosin α [9], which showed that the pH-dependent conformational change observed was completely reversible. IDPs show varying degrees of sensitivity to pH, which has mostly been attributed to the amount and position of charged amino acids. Charged amino acids have been a central focus when examining pH sensitivity as the current hypothesis suggests that the gained structure is the result of neutralization of the charged amino acid side chains [3]. IDPs generally have a high net charge, which leads to electrostatic repulsion between similarly charged amino acids. At extreme pH, the side chains are neutralized, and the IDP is free to adopt a more compact conformation. Based on this hypothesis, several studies have correlated a higher net charge with a higher degree of pH-dependent change or compaction [10,11]. However, the impact of uncharged residues has not been widely studied.

The objective of this study was to understand more about how the intervening sequence between charged residues impacts the pH-dependent change in intrinsically disordered proteins. To study this, the poly-E motif from the disordered PEVK region of the muscle protein titin was used as a model system. Titin is one of the three main filaments present in striated muscle, spanning half a sarcomere and connecting the thick filament to the thin filament. It is thought that it acts as a molecular spring and plays an integral role in passive tension and muscle contraction [12]. The PEVK region of titin has been shown to exhibit many of the characteristics associated with intrinsic disorder [13]. Two main motifs have been identified in the PEVK region as follows: PPAK and poly-E [14]. The PPAK motif is about 28 amino acids long, positively charged, and often starts with the amino acids P, P, A, and K. The poly-E motifs are negatively charged, mainly due to the presence of glutamate clusters. There is a high degree of alternative splicing of PEVK, resulting in PEVK regions with a different amount of poly-E and PPAK motifs based on muscle type and organism. 

The characteristics of PEVK make it a good model system for this study. Similarly to many intrinsically disordered regions, the poly-E motif has a high net charge. Compared to globular proteins, intrinsically disordered proteins and regions are typically rich in proline, glutamate, and lysine [3,15], which are the main amino acids in PEVK. In addition, clusters of negatively charged amino acids, specifically glutamates, have been identified as a motif present among IDPs [16], and these are the source of PEVK’s pH sensitivity. Therefore, PEVK, and more specifically, the poly-E motif sequence, is an ideal model system for understanding the pH sensitivity of IDPs.

These studies will also have functional significance since titin experiences pH shifts when the muscle fibers undergo acidification upon sustained activation [17]. However, the muscle is not the only microenvironment where pH fluctuations occur. With the advancement of technology, more methods for determining the pH of different environments have arisen. A prominent example of this is the pH in and around cancer cells. The extracellular microenvironment of tumors has been found to be acidic, with the pH ranging from 5.5 to 7.0 [18,19,20], while the intracellular pH is slightly alkaline [18,20,21,22]. A better understanding of the sequence determinants of pH sensitivity would allow for better predictions of IDP structures and functions in such environments. In addition, this knowledge could be used in the design of pH-sensitive peptides for a variety of applications, including drug delivery. 

For this study, a total of seven peptides were designed to test the effect of uncharged amino acids on IDR’s pH sensitivity, which included a reference sequence (similar to the wild-type poly-E) and six variants. In each of these variants, the net charge per residue and distribution of the glutamic acids were kept constant while changing the intervening sequences in order to isolate the effect of the uncharged amino acids. These linking amino acids had various degrees of bulkiness, hydrophobicity, proline content, and level of disorder. The pH sensitivity of these peptides was then examined using a combination of circular dichroism, size-exclusion chromatography, and molecular dynamics simulations. All of the tested peptides exhibited some degree of conformational change as a result of the pH, but two particular sequence characteristics most greatly impacted the pH sensitivity. The addition of aromatic residues led to a more compact and pH-sensitive peptide, while the removal of proline led to increased conformational flexibility. We also used 1H-NMR to confirm our hypothesis that a decreased pH resulted in increased conformational flexibility using the 28-amino acid peptide from which these peptides were derived. Overall, this work supports the hypothesis that changes in uncharged amino acids can result in differences in a peptide’s response to pH shifts. 

## 2. Materials and Methods

### 2.1. Reagents

All the Chemicals Used Were Purchased from Standard Chemical Suppliers such as Fisher Scientific.

### 2.2. Peptides

**Figure 1:**
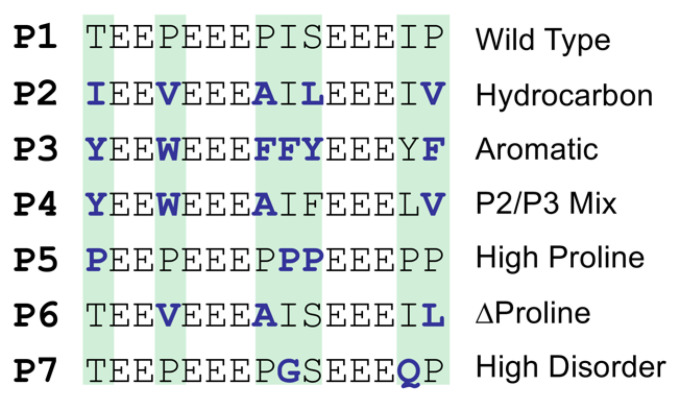
Sequences of the designed poly-E peptides. The reference sequence (P1) and the six variants are shown, with deviations from the reference highlighted in bold text.

The peptides used for this study were synthesized by the CEM Corporation (Matthews, NC, USA) and purified to >90% purity, as determined by UPLC. The peptide sequences are shown in Figure 1. The peptides were stored as lyophilized powders and rehydrated for each experiment. 

### 2.3. Circular Dichroism (CD)

Peptides 1, 5, and 7 were dissolved in Milli-Q water at a concentration of 8 mM, while the remaining peptides (2, 3, 4, and 6) were dissolved using 45% NH_4_OH at pH 8 due to their insolubility in water. All peptides were then diluted to a final protein concentration of 80 μM using a 20 mM potassium phosphate and 150 mM KCl buffer at different pHs. The pH of the sample was measured using a micro pH probe (Vernier, Beaverton, OR, USA), and, then, the samples were incubated at about 25 °C for at least 1 h before analysis. The CD measurements were taken on a JASCO J-1500 circular dichroism spectrophotometer (Jasco, Easton, MD, USA) using a 1 mm quartz cuvette. Each spectrum was collected by 3 accumulations using a 1 nm bandwidth and a 1 nm data pitch. Data points were collected in triplicate, buffer subtracted, and converted to molar ellipticity.

### 2.4. Size-Exclusion HPLC (SEC)

The samples were rehydrated in a 20 mM potassium phosphate and 150 mM KCl buffer at a range of pHs for a final protein concentration of 1 mg/mL. The samples were incubated at 25 °C for at least 1 h before the measurement. A TOSOH TSKgel G2500PWXL column was used for the separation, with the column being equilibrated with buffer prior to use. Each sample was run in triplicate, and the signal at 215 nm was used to determine the retention time. The samples were run on a Waters 2695 Separation Module equipped with a Waters 2996 Photodiode Array Detector (Milford, MA, USA). 

### 2.5. Computational Methods

Each peptide was simulated using implicit solvent constant pH molecular dynamics, as implemented in Amber [23,24]. The starting peptide structures were built as linear chains using the AmberTools utility tLEaP. All simulations employed the Generalized Born implicit solvent model with an effective salt concentration of 0.1 M and an effectively infinite cutoff of 1000 Å for force calculations. The constant pH algorithm allows protonation state changes for aspartate, glutamate, histidine, cysteine, lysine, and tyrosine residues, as described by the original constant pH protocol [24], while other amino acids are described by the Amber ff10 force field for proteins. The covalent bonds involving hydrogen were held rigid with the SHAKE algorithm. The general protocol entails minimization, heating, equilibration, pH equilibration, and production. The starting structures were first minimized for 100,000 total steps, where the first 10 steps employed the steepest descent and the remainder a conjugate gradient. During the minimization, no changes to the protonation states were attempted. Following minimization, the peptides were heated from 10 to 300 K in 1 ns without attempting protonation state swaps. The Langevin equations of motion were integrated with a 2 fs time step and a collision frequency of 5 ps^−1^. This was followed by an additional 1 ns of equilibration with constant protonation states. Next, we equilibrated the peptide at each pH from 0 to 12 with an interval of 1. We first equilibrated at a pH of 7. The final configuration was used as the starting configuration for simulations at pH 6 and 8. Each final configuration was then used for the next pH, either 5 or 9, respectively. This was progressively continued until simulations had been initiated at each pH. These simulations were 80 ns long with a 2 fs time step at 300 K. Swaps of the protonation states were attempted every integer number of steps closest to 50 steps per protonatable amino acid in the peptide (for example, for a peptide with 8 protonatable amino acids, swaps were attempted every 50/8 steps, or, in other words, every 6 steps). This equilibration protocol was designed to be much longer than the time required for the protonation states to converge, which Mongan et al. reported to occur within a few nanoseconds [24]. These much longer times were chosen to allow for the relaxation of the peptide configuration once the protonation state converged. We checked for equilibrium by observing the plateau of the potential energy and by comparing the three replicates for each system. The results we report were obtained from 200 ns production simulations. This protocol was completed in triplicate for each peptide system for estimation of error. The computational results are, therefore, based on approximately 55 μs of production simulation or 75 μs of equilibration and production simulation. 

The conformational state of the peptides is based on the peptide mass-weighted radius of gyration, residue secondary structure assignments, and amino acid side chain distances. The analysis is primarily calculated with the AmberTools utility cpptraj, but post processing is performed with inhouse scripts. The radius of gyration serves as a simple metric for measuring the compactness of the peptides, with small values indicating more compact or folded conformations and large values representing extended, unfolded conformations. We quantified the elongation of each peptide with an elongation ratio, which we define as the average radius of gyration at high pH (pH 10, 11, or 12) divided by the average radius of gyration at low pH (pH 0, 1, 2). We then used the Pearson correlation coefficient to statistically evaluate the relationship between the composition of the peptide and the elongation ratio. The correlation coefficient is calculated as the covariance of the two data sets divided by each standard deviation. Secondary structures are assigned for each amino acid at every frame using the DSSP algorithm. For the assessment of the degree of the structure, all structure assignment types are summed. The distances between the amino acids are calculated between the mass-weighted center-of-mass of each side chain.

### 2.6. NMR Spectroscopy

A 10 mg/mL stock of the 28-amino acid peptide that was used in previous studies [25] was prepared by dissolving the lyophilized peptide in ddH_2_O. The samples were prepared by diluting the peptide stock solution to 1:10 with a 10 mM KPO_4_ and 150 mM NaCl solution that was adjusted to the desired pH. The final samples were prepared by adding 10% D_2_O to the 1 mg/mL samples. The NMR data was collected on a 400 MHz JEOL magnet with a Royal probe using Watergate. A total of 6000 scans were collected over 10 hours at room temperature with a 4-second relaxation delay.

## 3. Results

Previous work in the Gage lab [25,26] has shown that the poly-E motif from the muscle protein titin exhibits structural changes as a function of pH. These changes are thought to occur as a result of charge repulsion from the glutamic acid clusters. While the role of glutamic acids in this pH sensitivity is understood, the function that the intervening sequences play is not well elucidated. Therefore, we developed a set of peptides based on the wild-type poly-E motif used in previous experiments with the same glutamic acid clusters but with varying intervening sequences.

The P1 peptide was based on the poly-E motif in the muscle protein titin and the longer 28-mer poly-E peptide from a previous publication [25]. The remaining peptides were derived from the P1 sequence with the same number of charged amino acids (glutamates) but with different properties for the intervening amino acids. The peptides P2–P4 contain different bulky amino acids. The bulkiness was determined using the Zimmerman method [27], where bulkiness is defined as the ratio of side chain volume to length, where values higher than 18 Å were considered to be bulky. According to this method, the bulkiest amino acids are W, V, I, L, F, and Y, in that order. These bulky amino acids can be divided into hydrocarbon and aromatic groups, which led to the design of the peptides P2, P3, and P4. The proline content is also an important factor in IDPs; therefore, variants with high proline content (P5) and no proline content (∆proline, P6) were designed. Lastly, the level of disorder was examined using a high-disorder peptide (P7), which was created by replacing any order-promoting residues with disorder-promoting residues. An amino acid was considered order- or disorder-promoting based on the TOP-IDP scale, which classifies the amino acids into these groups based on their prevalence in IDPs [15]. 

### 3.1. Peptides Show Structural Changes Based on pH

**Figure 2:**
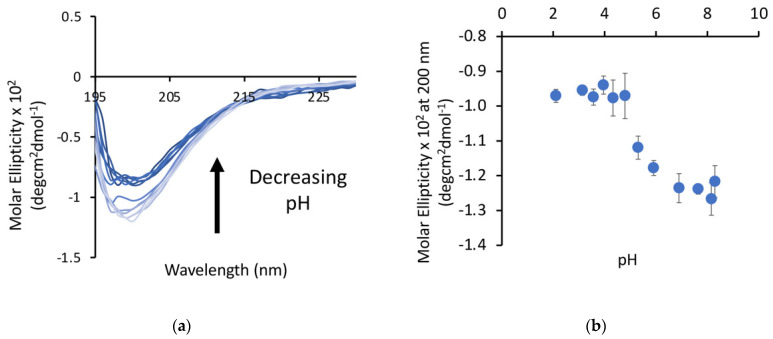
A peptide derived from the native poly-E sequence of titin shows a pH dependence as measured by CD. (**a**) CD spectrum of the poly-E peptide with an arrow indicating the direction of the change in the spectra with decreasing pH. (**b**) Molar ellipticity at 200 nm plotted against pH shows a transition to a more ordered state below pH 5.

Our previous work with poly-E sequences used longer sequences (28+ amino acids), so we first wanted to verify that shorter (15 amino acid) peptides exhibited similar behavior. The P1 peptide was dissolved in water and samples were prepared over a pH range from 2 to 8. The minima at 200 nm, as measured by circular dichroism (CD), decreased as a function of pH (Figure 2) in an apparent two-state transition, with a more structured state at low pH and a more disordered state at high pH. The transition between these states occurs between pH 4.5 and pH 6 for this peptide, which is higher than the pKa of free glutamic acid, but this matches the range for this transition observed in our other studies. This is consistent with a decrease in the random coil character of the peptide, suggesting that there may be some structure formation occurring at lower pH. 

### 3.2. Circular Dichroism Shows Differences in Peptide Structure at pH 7

**Figure 3:**
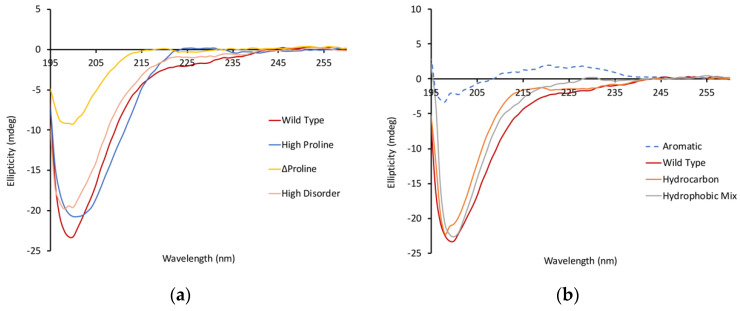
Peptides showing disordered characteristics at pH 7. CD spectra were collected for each peptide at pH 7 as a baseline. The majority of the peptides exhibited the characteristic 200 nm minima associated with a disordered structure. The ∆proline peptide (**a**) has a reduced minimum, suggesting that this peptide might have a small degree of ordered structure. The aromatic peptide (**b**) did not show any minima at 200 nm. The concentration of all peptides is 80 µM.

The focus of this study is to understand the pH sensitivity of these peptides; therefore, we wanted to establish a baseline for each of the peptides at neutral pH. The majority of the peptides have a similar degree of random coil structure as the wild-type (P1) peptide, with a minimum at 200 nm of around −20 mdeg (Figure 3a). However, peptide P6, which did not contain any prolines, had a minimum at 200 nm that was significantly smaller (~10 mdeg). 

**Figure 4:**
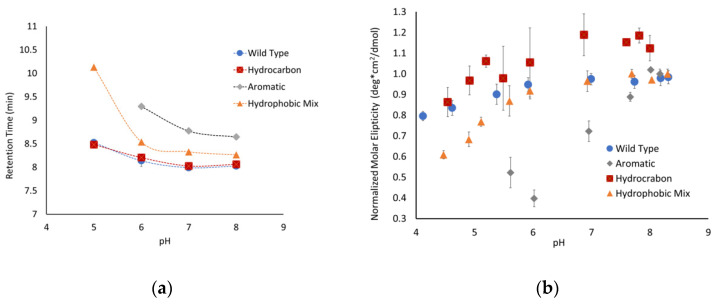
Conformational changes in peptides with hydrophobic linkers observed using (**a**) size-exclusion chromatography and (**b**) circular dichroism. Circular dichroism values at 200 nm were taken and normalized to the highest pH point for the peptide.

Even more dramatic, the aromatic peptide (P3) had a completely unique spectrum with no apparent disordered characteristics (Figure 3b) and a broad positive peak centered around 225 nm. This suggests that this peptide may have very few disordered characteristics, and the peak at 225 nm could be indicative of a polyproline type II helix structure in this peptide. Another possible explanation for the unique structural characteristic of this peptide is aggregation. To evaluate this possibility, we determined the retention time of this peptide by size-exclusion HPLC. The retention time of the P3 peptide was actually slower than that of the P1 peptide (Figure 4a), which is consistent with this peptide being in a more compact structure and not being aggregated.

### 3.3. Aromatic Amino Acids in Linkers Result in More Compact Conformations 

To further explore the unique structural state observed for the aromatic peptide, size-exclusion chromatography experiments were performed at a range of pHs. At pH 7, the peptide where all the linker regions contained hydrocarbon amino acids (P2) and the peptide with both hydrocarbon and aromatic amino acids (P4) both had retention times similar to the parent P1 peptide (Figure 4a). The P4 peptide does have a retention time that is slightly slower than the P1 peptide, suggesting that this peptide might have a slightly more compact structure. In contrast, the aromatic-containing peptide (P3) has a retention time that is almost a minute slower than the P1 peptide, which is consistent with a significantly more compact structure. The linker regions in the P3 peptide consist completely of aromatic amino acids, while the linkers in P4 are ~40% aromatic. This difference in amino acid composition is consistent with the degree of shift in the pH 7 retention time and suggests that the incorporation of aromatic residues helps to compensate for the charge repulsion effects of the glutamate clusters, allowing for a more compact structure.

The peptides were separated over a range of pHs to determine how changes in the charge of the glutamates impacted the conformation of the peptides. The P2 peptide, with the hydrocarbon linker regions, mirrored the behavior of the native P1 peptide. This suggests that increasing the number of hydrophobic side chains from amino acids such as leucine and valine was not sufficient to alter the peptide’s extended conformation due to charge repulsion. The P1 and P2 peptides exhibited a slow increase in retention time as the pH dropped, which is consistent with the peptide slowly gaining flexibility and becoming more compact, though this is not a very significant change over the pH range that was tested.

The two peptides with aromatic amino acids showed much more conformational flexibility. The P3 peptide started with a significantly higher retention time that shifted over the pH range that was tested. The P4 peptide mirrored the behavior of the P1 and P2 peptides but exhibited a significant shift at pH 5. Similar behavior was observed by CD for the P3 and P4 peptides, having significantly shifted molar ellipticities compared to the control (Figure 4b). One challenge with analyzing the data for the P3 peptide is that it does not have a pronounced minimum at 200 nm, resulting in much more scatter in the data. It is also important to note that a full range of pH data could not be obtained for all peptides due to their decreased solubility at lower pHs. Even with the limited data, it is clear that the inclusion of aromatic amino acids alters the conformational flexibility of the peptides with high degrees of charge repulsion.

### 3.4. ΔProline Peptide Exhibits More pH Sensitivity

**Figure 5:**
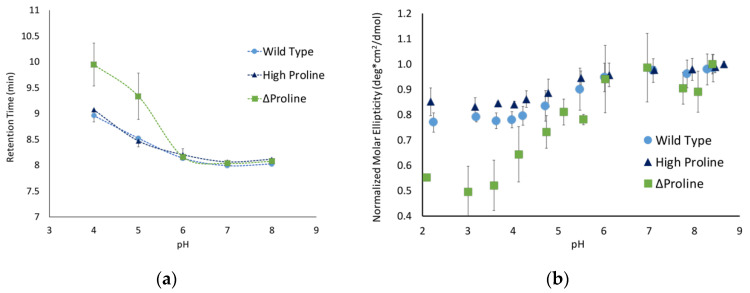
Effects of proline on pH sensitivity as observed with (**a**) size-exclusion chromatography and (**b**) circular dichroism. Circular dichroism values at 200 nm were taken and normalized to the highest pH point for the peptide.

Proline is one of the primary amino acids in the PEVK region of titin, which is prevalent in intrinsically disordered proteins and is known to disrupt secondary structures. These factors suggest that the proline content might modulate the conformations of peptides with a high degree of charge repulsion. To test this hypothesis, we developed the following two peptides with altered proline content: a high-proline variant (P5), with only prolines in the linker region, and a ∆proline variant (P6), where the three prolines were replaced by valine, alanine, and lysine. The high-proline variant behaved similarly to the P1 peptide (Figure 5), indicating that the increased proline content above the native sequence did not influence the conformational flexibility. The Δproline peptide exhibited similar behavior as the P1 and P5 peptides between pH 8 and 6, but showed a significantly higher pH sensitivity below pH 6. The retention time for P6 was ~1 minute slower below pH 6, and the molar ellipticity shifted toward a less disordered state. Taken together, this suggests that the removal of proline and replacing it with other similarly sized but less rigid amino acids allowed the peptide to adopt a more compact conformation.

### 3.5. Wild-Type Poly-E Peptide Shows Most Significant Compaction with SEC at pH 3

**Figure 6:**
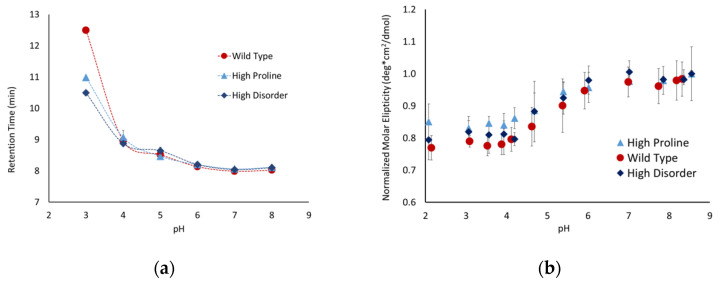
pH-dependent conformational changes as observed with (**a**) size-exclusion chromatography and (**b**) circular dichroism. Circular dichroism values at 200 nm were taken and normalized to the highest pH point for the peptide.

It was only possible to perform SEC and CD experiments on three of the peptides at pHs below the pKa of free glutamic acid due to the solubility of the peptides at that pH range. These three peptides; the wild-type, high-proline, and high-disorder, exhibited similar behavior in both SEC and CD experiments until pH 3, where the wild type showed a higher degree of pH-dependent compaction (Figure 6). It is interesting to note that this was only observed in SEC experiments, as there was no observable difference in the 200 nm molar ellipticity for any of these peptides at pH 3. This suggests that, while the addition of proline or disorder-promoting side chains influences the overall compaction of the peptide, it does not alter the secondary structure being detected by CD. 

### 3.6. Addition of Hydrophobic Amino Acids Increased Precipitation

**Figure 7:**
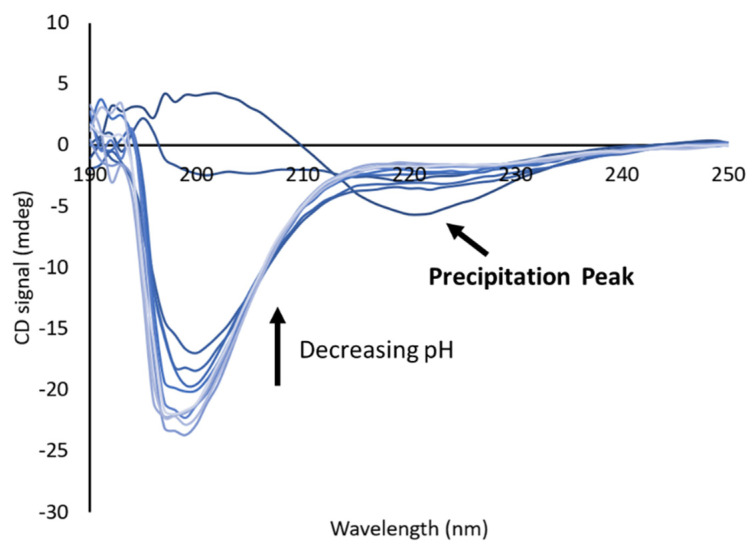
Hydrocarbon peptide, which precipitates at low pH, as seen by CD. The trough at 220 nm corresponds to pH 2.

As mentioned previously, not all peptides were soluble in the entire pH range tested. While the wild type and the peptides with additional proline or disorder-promoting residues (P1, P5, and P7) were soluble in water, the peptides with additional hydrophobic residues displayed limited solubility at lower pHs. This was observed through both a visible precipitate and by using CD, since the precipitation of the peptides was accompanied by a change in the CD spectrum, which is characterized by the absence of the significant random coil behavior and the presence of a broad trough at around 220 nm (Figure 7). This effect was seen in multiple peptides, but the representative data shown in Figure 7 are for the hydrocarbon variant (P2). The appearance of this peak helped to identify when aggregation that was not visible was occurring, and samples containing this peak were not included in the data analysis.

Interestingly, this occurred for the hydrocarbon variant but not the Δproline variant, despite the two having similar sequences. The Δproline variant, however, did contain an additional tyrosine and serine that the hydrocarbon did not, which suggests that the added polar groups helped with solubility at low pHs. In addition, the pH at which this insolubility occurred increased with the addition of aromatic amino acids, with the lowest soluble pH being at around pH 4.5 for the hydrophobic mix peptide and around pH 5.5 for the aromatic peptide.

### 3.7. Computational Modeling Shows Similar pH-Dependent Shifts

**Figure 8:**
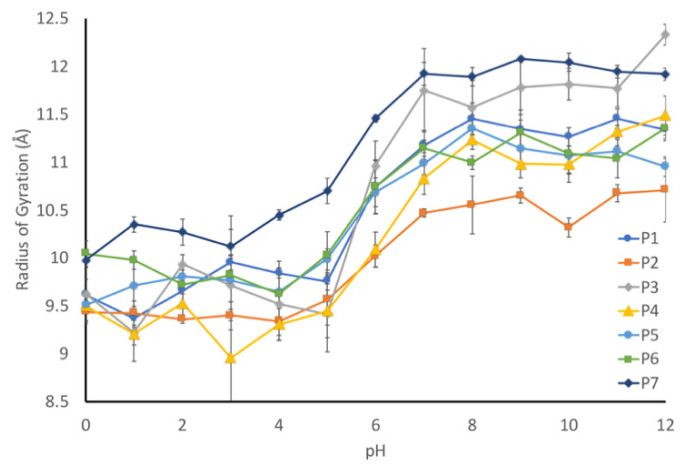
pH-dependence of the average radius of gyration for each peptide studied. All peptides are found to elongate with pH. Some show an essentially linear trend (for example, P2), while others have a pH where there is a distinct “switch” to longer configurations (such as P3).

We applied molecular dynamics simulations to probe the nature of the pH-driven conformational changes. First, the radius of gyration for each peptide was computationally determined to validate that our modeling parameters could reproduce our experimental results. Each sequence was simulated using constant pH molecular dynamics to determine the radius of gyration at a given pH, which was plotted against pH (Figure 8). As it can be seen, all seven sequences exhibited an increased radius of gyration (indicating a more elongated structure) at the higher pH range, with a transition between pH 4 and 7. To help quantify this transition, we calculated an elongation ratio (Table 1), which was calculated by dividing the radius of gyration at high pH (10–12) by the radius of gyration at low pH (0–2).

The elongation ratio highlighted a couple of aspects of the relationship between the sequence and the pH. First, the protonatable residues have a strong effect on the elongation ratio. The correlation coefficient is 0.8, which indicates that the more charged residues at low pH will result in a more elongated peptide. Second, the elongation ratio is negatively correlated with the number of prolines (correlation coefficient of −0.2). This means that the inclusion of proline residues will slightly decrease the elongation effect.

The simulation is consistent with our experimental data, but the simulations did not seem to capture some of the more nuanced experimentally observed behaviors. For example, the P6 (∆proline) peptide exhibited a much higher degree of pH sensitivity on SEC, but this was not observed in the modeling. This suggests that the molecular dynamics simulations are more consistent with the CD data than with the SEC data.

**Figure 9:**
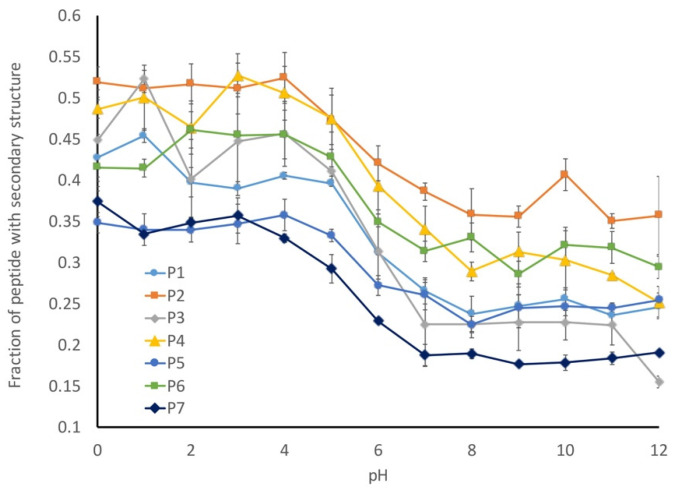
pH-dependence of the fraction of secondary structures for each peptide studied. The amount of secondary structures decreased with the increasing pH, which is consistent with the formation of an elongated conformation.

Our working hypothesis is that the conformational flexibility increases as the pH decreases due to a decreased charge repulsion, which is supported by the increased radius of gyration at lower pHs. Next, we looked at the number of amino acids that have assigned secondary structures in our models to determine the fraction of the peptides with secondary structures (Figure 9). This analysis was inversely correlated with our radius of gyration (R_g_) calculation, with more secondary structures being observed in the peptides with lower R_g_ values. This suggests that the preferred conformational state for these peptides is a more collapsed state and that they can form limited secondary structures in the absence of any charge repulsion.

While all of the seven peptides underwent a conformational shift over the same pH range, the magnitude of change was not consistent. The wild-type peptide sequence (P1) underwent a 20% change in secondary structure between pH 1 and pH 11, and a similar behavior was observed for the P4 peptide, which was a mix of hydrocarbon and aromatic amino acids. In contrast, the two peptides with the altered proline content (P5 and P6) only exhibited a 10% shift in secondary structures, while the P2 (hydrocarbon-containing) and P7 (disorder-promoting) peptides exhibited a 15% change in secondary structures. In general, any alterations that were made to the liner sequence resulted in a decreased shift in secondary structures relative to the wild-type peptide sequence.

**Figure 10:**
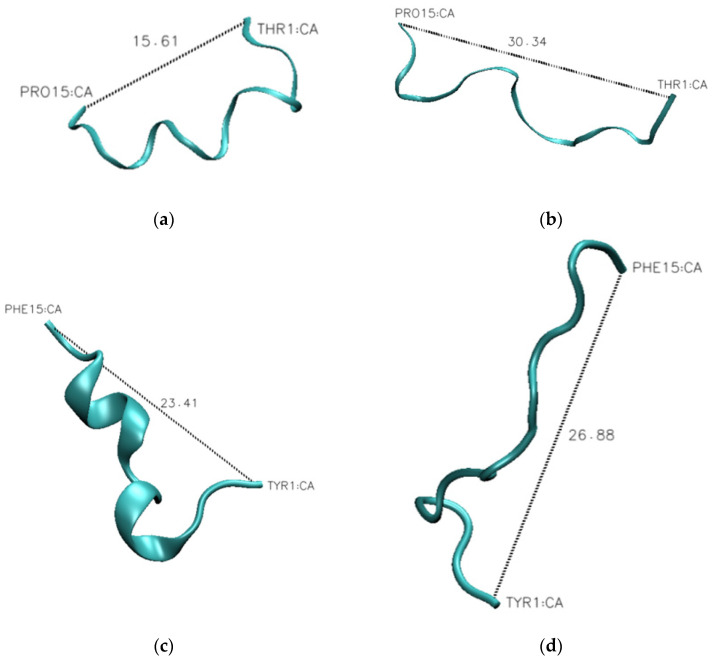
Representative structures of the wild-type peptide (P1) and the aromatic peptide (P3) show an increased secondary structure in P3. Representative structures of the P1 peptide at pH 1 (**a**) and pH 12 (**b**) demonstrate that the P1 peptide has a more extended conformation at pH 12. Representative structures of the P3 peptide at pH 1 (**c**) and pH 12 (**d**) demonstrate that the P3 has a significant secondary structure at pH 1. The distance between the initial and final α-carbons are reported in ångströms.

**Figure 11:**
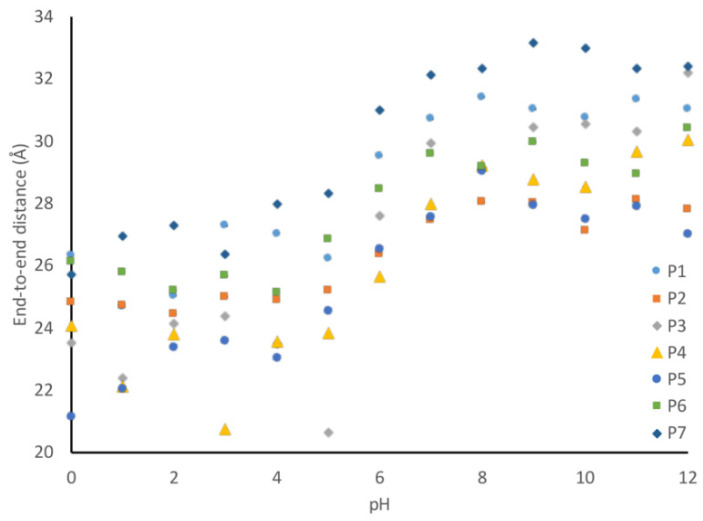
Average end-to-end distances for each peptide. End-to-end distance is defined as the center-of-mass distance between the first and last amino acid in the peptide. Errors in the mean are reported for each data point but are smaller than the symbols. This data show that the peptides all extend when the pH increases.

The one outlier to this observation is peptide P3, which contains aromatic amino acids in the linker sequences. This peptide exhibits a 30% change in secondary structure over the evaluated pH range, which is 10% larger than that observed for the wild-type peptide. This difference becomes evident when looking at the representative structures of the peptides at each pH (Figure 10). The P1 peptide is in a more collapsed conformation at pH 1 (Figure 10a) than at pH 12 (Figure 10b). A similar collapse is observed with the P3 peptide (Figure 10c,d), though the P3 peptide shows evidence of a helical structure at low pH (Figure 10c). What differentiates these peptides is that peptide 3 has a higher aromatic content, and as aromatic amino acids have a tendency to interact through Pi-stacking, this could have led to the increased predicted secondary structure observed at low pH. To further support this hypothesis, we looked at the average distance between each of the aromatic amino acids at high and low pH. While we do not see an alpha-helical content in our CD spectra for the wild-type peptide, it is likely that we are capturing a transient helical structure in the modeling that is not stable enough to be observed by CD. The unique behavior of this peptide in MD simulations does provide an explanation for why it exhibits such a unique spectrum in CD experiments. Further, the elongation shown in Figure 10 can be quantified by evaluating the average end-to-end distance of each peptide at every pH. Using these end-to-end distances, it can be seen that all peptides become more compact at low pH (Figure 11).

To further test the hypothesis that the aromatic amino acids are closer at lower pH, we looked at the average distance between each of the aromatic amino acids at high and low pH. There are 21 different aromatic–aromatic pairings in the P3 peptide, and of these 21 pairings, 16 of them showed closer average distances at lower pH. For example, W4 and Y14 became 8.6 Å closer at low pH. Similarly, Y10 and Y14 shifted closer by 3.3 Å under low pH conditions. This is consistent with our model that the increased flexibility at lower pH due to the decreased charge repulsion allows for an increased interaction between aromatic amino acids. 

### 3.8. ^1^H-NMR Spectra Exhibit More Compact Structure at Lower pH

We used ^1^H-NMR to further validate that the observed shifts in HPLC and CD are due to the peptide assuming a more compact structure. These experiments were conducted using the 28-amino acid peptide sequence used to derive the peptides utilized in this study. This peptide was used since it had higher solubility than the 15-amino acid peptides and a higher molecular weight, so the structural transitions would be more apparent. Both the 28-amino acid and the 15-amino acid peptides exhibit similar patterns of behavior in CD and HPLC; therefore, data collected with one system can be extrapolated to the other system [25].

**Figure 12:**
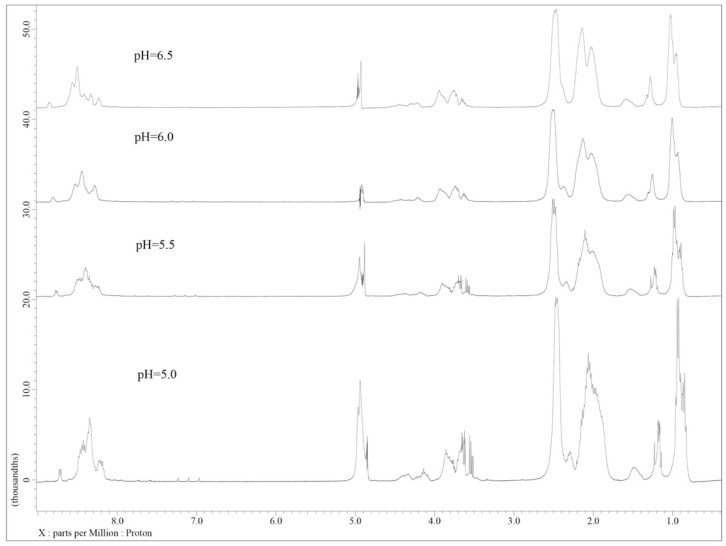
^1^H-NMR spectra of the poly-E peptide at varying pHs, showing increased compaction at lower pHs. Spectra of our 28-amino acid peptide were collected at intervals of 0.5 pH units from pH 5.0 to pH 6.5, which spans the pH range where conformational transitions are observed in both CD and HPLC [25]. As it can be seen, the peaks in the methyl and aliphatic regions of the spectra become sharper, which is consistent with a more compact structure.

Samples were generated by preparing a 10 mg/mL stock of the 28-amino acid peptide in ddH_2_O, which was then diluted to 1:10 using potassium phosphate solutions that were adjusted to the desired pHs. This approach was used to ensure that there was an equivalent concentration of the peptide in each sample. A total of 6000 scans were collected on each sample to minimize background noise and to compensate for the low sample concentrations. When the spectra were overlaid (Figure 12), there were apparent differences in the pH 5.0 and 5.5 spectra compared to the pH 6.0 and 6.5 spectra. Firstly, there is an increase in the signal from the backbone amides (~8 ppm) as the pH decreases, which is consistent with the lower rate of amide exchange that occurs at lower pHs. This is a validation of the differences in pH in these samples. Secondly, and more importantly, the peaks associated with the methyl and aliphatic functional groups (~1–4 ppm) are sharper at lower pHs, with the transition occurring between pH 5.0 and 6.0, which is consistent with the range over which shifts have been observed by both CD and HPLC for this peptide. This is further evidence that the decreased 200 nm minima observed in CD and the increased retention time observed in HPLC are due to the peptide adopting a more compact structure at lower pHs.

## 4. Discussion

Many intrinsically disordered proteins are sensitive to changes in pH and undergo conformational changes as a result of the protonation or deprotonation of their charged amino acids. One such disordered region is the poly-E motif found in the muscle protein titin. While it is known that the clusters of charged glutamate residues play an important role in the level of pH sensitivity of this motif [25], the role of the uncharged amino acids in modulating pH sensitivity is unclear. In this study, we designed a set of peptides to understand the role of the amino acids in between the charged glutamate clusters, based on the poly-E motif used in previous investigations [25]. In each peptide, the position and amount of charged residues were kept consistent, while the intervening amino acids were altered to have different properties. In total, seven peptides were tested, a wild type based on a poly-E sequence found in titin and six variants. The effect of adding hydrophobic amino acids was tested using peptides with hydrocarbon side chains (P2), aromatic side chains (P3), and a mix of different types of hydrophobic amino acids (P4). The effect of adding and removing proline was also examined with a Δproline (P6) and a high-proline (P5) peptide. Lastly, the effect of adding disorder-promoting residues was examined using a peptide (P7) where all order-promoting residues were replaced.

The effect of pH on these peptides was tested using circular dichroism and size-exclusion chromatography. All seven peptides showed a decrease in random coil character by CD and molecular dynamics as the pH was lowered, suggesting that the decreased pH reduced the charge repulsion, allowing some degree of structure to form. Most of the peptides exhibited a two-state transition in CD, with a more ordered low pH state and a more disordered high pH state. In a similar pattern, the longer retention times at lower pH were measured via SEC for each peptide, indicating a shift to a more compact state. This is consistent with the glutamic acids becoming protonated and neutralizing their charges, and with the decreased charge repulsion, allowing the peptides to fold into a more compact state. 

### 4.1. Aromatic Interaction

The introduction of aromatic amino acids resulted in an interesting difference in peptide behavior. The peptides containing aromatic amino acids (P3 and P4) exhibited longer retention times at neutral pH compared to the wild-type peptide. This suggests that these peptides have a more compact conformation, even at neutral pH. The aromatic peptide (P3) showed the greatest degree of compaction at pH 7, but compaction was also observed for the peptide with linkers consisting of both aromatic and hydrophobic amino acids. Our working hypothesis is that the increase in the number of aromatic residues increases the magnitude of compaction. As the hydrocarbon peptide did not exhibit these differences, it is likely that the aromatic character and not the hydrophobicity of the peptides led to this increased compaction. 

The CD spectrum of the aromatic peptide was significantly different from the wild type, with a minimal random coil character and a peak at 220 nm that could be indicative of a polyproline-type helix character. The P3 peptide also exhibited the largest degree of compaction by HPLC over the pH range that could be tested. Finally, this peptide was observed to have the largest elongation ratio from the molecular dynamics, indicating a significant compaction with decreasing pH. This behavior was not seen in the hydrocarbon variant or the hydrophobic mix variant, which suggests that it is the association of multiple aromatic rings close to each other that is leading to this altered state, potentially through some sort of stacking interaction of the aromatic rings. This hypothesis is supported by our computational analysis, which showed that the aromatic amino acids in P3 are closer at low pH than at high pH. Similar behavior has been observed in other systems, such as in the VPg protein from the *Sesbania mosaic* virus, in engineered constructs used to study IDP phase separation, and in prion-like domains [28,29,30].

The peptide containing linkers with a mix of aromatic and hydrophobic amino acids showed an increased level of sensitivity to pH relative to the P2 and P3 peptides. SEC showed a significant shift in the retention time between pH 5 and 6, a range that only resulted in a moderate change for the wild-type and P2 peptides. Similar results were observed by CD, suggesting that the increased aromatic and hydrophobic content may create an environment that facilitates the protonation of the glutamic acids. This is further supported by the MD simulations, which showed a smaller shift in the elongation ratio and a change in the secondary structure content with changing the pH for P2 relative to P4. The higher degree of compaction observed with P4 relative to P2 is most likely due to the inclusion of the aromatic residues within the peptide, stabilizing a more compact structure through interactions between the aromatic amino acids.

### 4.2. Effect of Proline

Proline, the only imine acid, has several unique characteristics that differentiate it from other small amino acids with hydrophobic side chains. Due to the secondary amine, proline has a hydrogen bond donor but lacks an acceptor, resulting in a propensity to be solvent-exposed and less hydrophobic [31]. This and the increased extension observed in sequences that are high in proline [11] may contribute to proline’s disorder-promoting nature. It is classified as the most “disorder-promoting” amino acid as it is the most prevalent amino acid in IDPs [15]. In addition, proline content has been identified as an important factor when considering the compaction of an IDP, with increased proline content correlating to increased extension of an IDP [11]. 

To test the effect of proline on the poly-E construct, the following two constructs were designed: one in which all of the non-glutamate residues were replaced with prolines (P5) and one where all of the prolines were removed and replaced with small, hydrophobic amino acids, such as valine, alanine, and lysine (P6). This change resulted in a greater pH sensitivity of the Δproline peptide, especially at the lower pHs. This could indicate that the proline is inhibiting the collapse of the peptide, which aligns with previous knowledge that it is more energetically favorable for proline to be solvent-exposed [32], and that it promotes a more elongated conformation. This hypothesis is supported by the observation that the P5 peptide has one of the lower fractions of secondary structures in the MD calculations. The P5 peptide also has the smallest shift in the fraction of peptides with secondary structures at low pH. Interestingly, the P6 peptide, while having a higher fraction of secondary structure-containing peptides than the P5, also did not exhibit a significant shift in that fraction relative to the pH. 

### 4.3. Wild-Type Poly-E Is Optimized for pH-Dependent Change

The wild-type peptide showed the highest degree of pH-dependent compaction, as seen by the SEC data, when compared with the other peptides at pH 3. This is not entirely unexpected as the other two peptides observed at that pH were the proline-containing variant and the high-disorder variant. With more disorder-promoting residues, the high-disorder variant may not adopt as much of a structured, collapsed state upon pH change. The added glycine residue could provide flexibility that reduces any pH-dependent conformational change and creates more of an ensemble of conformations rather than one more compact state. The proline could have a similar effect as it has been shown to act as a secondary structure breaker [31]. It is possible that the other peptides, when compared to the wild-type, did not have enough hydrophobic character (or other factors) to drive the collapse at lower pH, despite the removal of some of the electrostatic repulsion by the protonation of the glutamic acids. It is important to mention, however, that this increased level of pH-dependent change was not observed in the CD or molecular dynamics data. This suggests that although the peptide is in a more collapsed state, this does not result in a significant difference in the amount of secondary structures. 

The wild-type peptide was taken from the poly-E sequence from the PEVK motif of titin. Titin is a protein present in the sarcomere that can become acidic when activated. After strenuous exercise, the muscle pH can drop as low as 6.50–6.87 [17] as a result of lactic acid and carbon dioxide production. While the conformational change for the peptide shown here occurs at a pH that is too low to be psychologically relevant, this does not preclude the possibility of this occurring in a full-length poly-E, as the increased length could lead to susceptibility at higher pHs. As the wild-type peptide showed the greatest change, it is possible that poly-E has been evolutionarily tuned to some degree to be sensitive to pH fluctuations.

### 4.4. Peptide Solubility

When designing peptides, it is important to consider the solubility of IDPs in the environment that they will be used in. Overall, this study showed that poly-E type peptides with intervening residues containing few or no polar groups have significant solubility issues, particularly in water and in buffer. The peptides that did not dissolve readily in water were the hydrocarbon, aromatic, hydrophobic mix, and ∆proline variants. The wild-type, high-proline, and high-disorder peptides dissolved readily in water. The fact that the high proline variant did not experience the same solubility issues as the other variants indicates that proline does not behave similarly to the other hydrocarbon-containing amino acids, despite containing no additional charged groups. This aligns with previous work, which indicates that proline has less hydrophobic character than its hydrocarbon counterparts [32]. It is also interesting to note that the presence of additional aromatic amino acids in the linkers decreased the pH range at which the peptide was soluble. The hydrophobic mix peptide, which only contained some aromatic amino acids, was soluble until around pH 4.5, while the aromatic peptide, which only contained aromatic amino acids in the linkers, lost solubility at around pH 5.

The insoluble peptides were able to be solubilized in DMSO, presumably due to its nonpolar methyl groups. However, even the low concentrations of DMSO interfered with our experiments, and, therefore, it could not be used as a solvent. If similar peptides are designed for physiological applications or assays, DMSO is not a viable solvent. As a result, care should be taken when introducing hydrophobic (especially aromatic) amino acids into such peptides if solubility in water or buffer is needed.

Overall, this study provides some interesting insights into the design of pH-responsive peptides and the different characteristics that can impact the pH-dependent conformation observed.

## Figures and Tables

**Table 1:** Elongation ratios for each peptide.

Peptide	Elongation Ratio
P1	1.19 ± 0.02
P2	1.12 ± 0.02
P3	1.25 ± 0.06
P4	1.20 ± 0.04
P5	1.14 ± 0.02
P6	1.13 ± 0.03
P7	1.17 ± 0.02

## Data Availability

All raw data is available upon request.
